# Chiral quantum optics with scalable quantum dot dimers

**DOI:** 10.1038/s44310-026-00148-y

**Published:** 2026-09-09

**Authors:** L. Hallacy, D. Hallett, A. Fenzl, N. J. Martin, R. Dost, A. Verma, J. Fletcher, I. Farrer, L. Antwis, M. S. Skolnick, L. R. Wilson

**Affiliations:** 1 School of Mathematical and Physical Sciences, University of Sheffield, Sheffield, UK; 2 School of Electrical and Electronic Engineering, University of Sheffield, Sheffield, UK; 3 Ion Beam Centre, University of Surrey, Guildford, UK

**Keywords:** Optics and photonics, Physics

## Abstract

We present a scalable method for electrically tuning multiple spatially separated quantum dots (QDs) embedded in photonic crystal waveguides. Ion implantation into the top p-doped layer of a *p-i-n* diode creates high-resistivity tracks, providing electrical separation between adjacent regions. This method preserves the guided-mode profile of the waveguide, minimising backscatter and loss, enabling the observation of highly directional emission from two individually addressable QDs coupled to a glide-plane photonic crystal waveguide. We demonstrate control of the detuning between the QDs, enabling measurements of different indistinguishable spin-state combinations of two highly directional QDs coupled to the same mode. Second-order photon correlation measurements provide a sensitive probe of the chirality-dependent photon statistics, which are in good agreement with a waveguide-QED master equation model. Our results mark an important step towards scalable, multi-emitter architectures for chiral quantum networks.

## Introduction

Chiral quantum networks offer a scalable route to distributed quantum information processing, enabling deterministic transfer of quantum states and photon-mediated entanglement between distant qubits^1,2^. Such architectures underpin applications ranging from programmable quantum processors^3,4^ and quantum simulators^5^ to fault-tolerant quantum repeaters^6^, where efficient and directional spin-photon interfaces form the key building block^7^. Realizing these networks requires platforms that combine near-unity coupling efficiency with strong spin-selectivity, while supporting scalable integration and fast tunability of multiple quantum emitters. Self-assembled QDs are highly controllable quantum emitters with high quantum efficiency in a solid-state platform^8–10^, making them ideal for building these systems.

Photonic crystal (PhC) waveguides offer several key advantages for this task. Their periodic dielectric structure supports slow-light modes that enhance light-matter interactions, simultaneously yielding large *β*-factors, strong Purcell enhancement, and high chiral contrast^11–13^. A glide-plane waveguide (GPWGs) is a particularly promising PhC platform^1,14,15^. In GPWGs, the intentional breaking of mirror symmetry enables circularly polarized emission from a QD to couple directionally into the guided mode. Regions of high chiral contrast spatially overlap with areas of strong electric-field intensity, supporting highly directional coupling when emitters are positioned at these optimal locations^16^ as well as the general benefits of PhC waveguides described above.

Recent comparative studies show that GPWGs outperform conventional W1 line-defect waveguides for achieving chiral coupling: while W1 structures typically yield only a small fraction of QDs with >80% chiral contrast, GPWGs exhibit the highest proportion of high-contrast QDs, with both simulations and experiments demonstrating robust regions where *β* ≳ 80% and chiral contrast exceeds 90%^17,18^. The low positional dependence of GPWGs is especially attractive for Stranski-Krastanov QDs, which exhibit inhomogeneous emission energies and random spatial positions^19,20^. These combined figures of merit make GPWGs uniquely suited for scalable chiral quantum optics, where both efficient photon extraction and deterministic spin-photon interfaces, coherently mapping the QD spin state onto a well-defined optical mode, are required.

Beyond efficient interfaces, chiral coupling enables deterministic few-photon nonlinearities^21–24^. Pairs of chirally coupled emitters can act as near-ideal photon sorters, separating single- and two-photon components with fidelities above 0.999 in ideal cases, enabling deterministic non-linear sign gates and Bell-state analysis^25^. Chiral coupling in chains of waveguide-coupled emitters forms a cascade quantum system that enables spontaneous entanglement generation^26^, robustness against emitter separation^27,28^, the formation of isolated emitter dimers^29^ and passive two-photon logic gates^30–32^. Hybrid nanocavity-waveguide platforms further enhance functionality, combining strong Purcell enhancement (*F*_*P*_ ≈ 11) with directional contrast ( ~ 88%) and wide-range electrical tunability, offering practical routes to electrically reconfigurable spin-photon interfaces^33,34^. While these advances establish a clear foundation for scalable chiral networks, achieving stable and predictable control over multiple emitters remains a central challenge that we address here.

Observing many-body effects requires control over the relative detuning between emitters. Electrical tuning of QD transition energies via the quantum confined Stark effect (QCSE) offers a powerful solution to build programmable circuits for chiral quantum optics. It provides fast, reversible, and localized control of QD emission with compact integration. Furthermore, it is the only approach that enables large-scale synchronization of remote emitters, real-time stabilization of interference, and potential for dynamic wiring of graph states in chiral networks^35^. However, existing demonstrations achieve independent tuning by physically etching through the *p*-doped layers of a *p-i-n* membrane, effectively splitting the device into two diodes^36,37^. When introduced into a photonic crystal, such etching perturbs the local group index and increases both backscattering and out-of-plane losses^38^.

In this work, we overcome these challenges by using ion implantation to provide electrical control of multiple emitters in a GPWG without compromising its advantages. Ion implantation provides lateral electrical isolation without physically etching the photonic crystal lattice, thereby preserving its optical properties. We extend on earlier demonstrations in photonic crystal nanocavities^39^ and waveguide systems where significant optical perturbations were introduced in prior work^40^, carefully tuning the implantation profile so as to maintain the optical integrity of the device while providing sufficient isolation.Using this approach, we demonstrate the control of individual QDs in a GPWG. Combining Stark tuning with Zeeman splitting, we selectively bring independently addressable spin states (*σ*^±^) from two QDs into resonance. By exploring photon correlations from different spin-state pairs, we demonstrate direction-dependent cooperative behaviour in pairs of photons emitted into the waveguide mode, highlighting the potential of ion-implanted waveguides as a platform for stable, electrically reconfigurable chiral spin networks.

## Results

### Photonic properties of the waveguide device

**Fig. 1:**
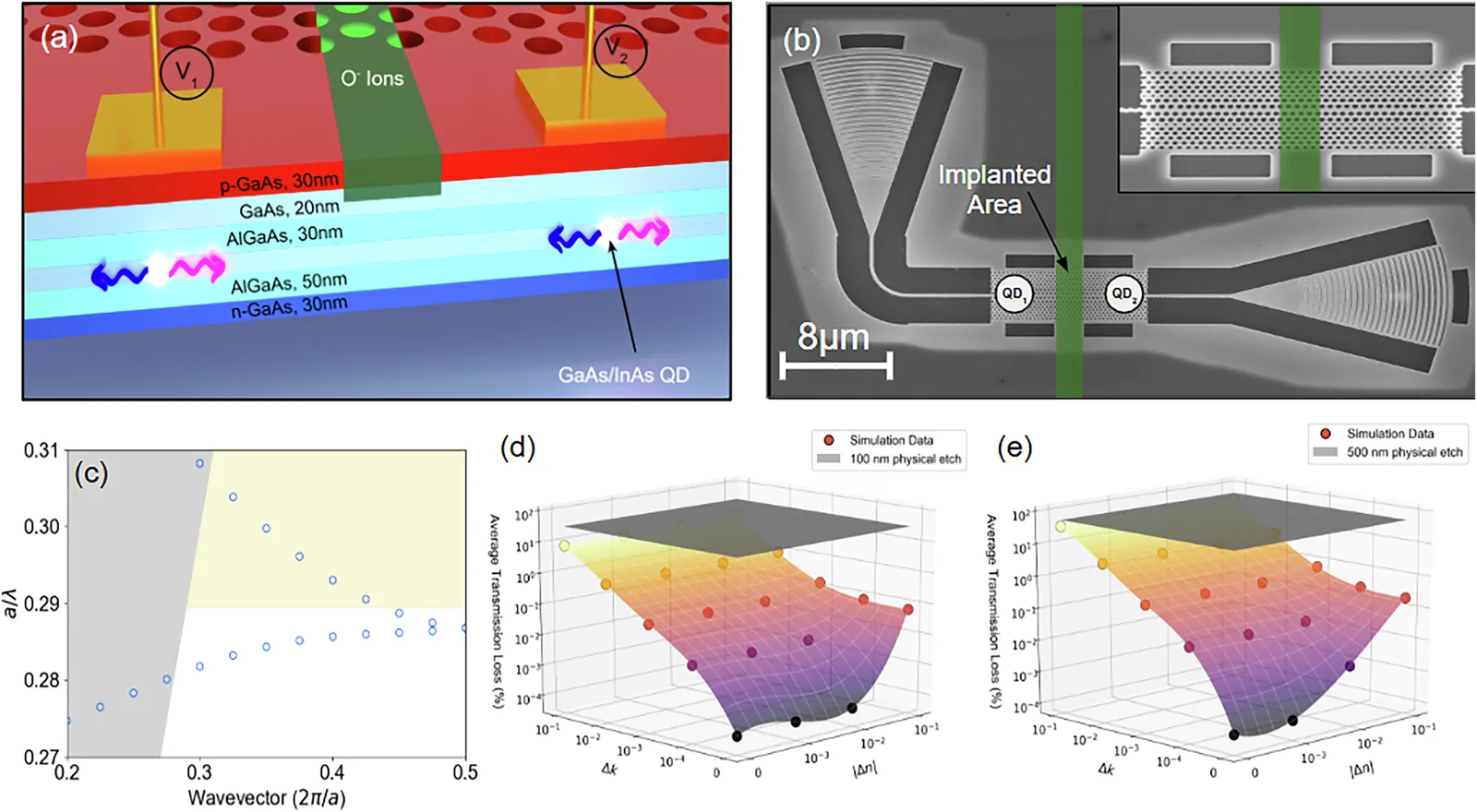
Device structure and implantation effects on optical properties. **a** Schematic cross-section of the *p-i-n* membrane structure used for ion implantation, where *p-i-n* denotes the p-type, intrinsic, and n-type diode layers where voltage (V) can be applied to different sections labelled 1 and 2. **b** Top-down SEM image of the GPWG, where the green pseudo-colour strip marks the 500 nm wide implanted region across the centre of the waveguide. QD_1_ and QD_2_ denote QDs located on the left- and right-hand sides of the implanted region, respectively. **c** Photonic band structure of the GPWG, plotted as normalized frequency, *a*/*λ*, against normalized wavevector, where *a* is the photonic crystal lattice constant and *λ* is the wavelength. The yellow shaded region indicates the guided band used for the optical simulations. Simulated transmission loss as a function of refractive-index change, *Δn*, and extinction-coefficient change, *Δk*, for implanted region widths of (**d**) 100 nm and (**e**) 500 nm. The circular markers show the simulation data, the coloured surface shows the interpolated transmission-loss response, and the grey plane shows the calculated loss for an equivalent physical etch.

The implantation technique is applied to a GPWG (wafer structure and SEM image shown in Fig. 1a, b)^15^, chosen for its broken mirror symmetry, which enables highly directional light-matter interactions. In these structures, spin-momentum locking allows the directional coupling of circularly polarized dipole transitions from quantum emitters into guided modes with near-unity efficiency^41^. This chiral coupling is particularly pronounced in the vicinity of the photonic band edge, where the waveguide supports slow-light modes with high group index (*n*_*g*_ ≫ 1). The slow-light region is highly sensitive to perturbations in the refractive index profile: even minor dielectric discontinuities introduced by fabrication imperfections or material damage can induce backscattering, mode disruption, and substantial radiative losses^38^. As such, the glide-plane waveguide serves as an ideal testbed for this method.

Ion implantation achieves decoupling of diode sections' electric fields by modifying the dopant profile in the *p*-layer rather than physically altering the structure. This process introduces charge traps that form additional defect states with energies lying below the near-valence states of the *p*-doped GaAs. This immobilizes the free carriers, pinning the local Fermi level^42^. This pinning effect prevents modulation of the local electric field under applied bias, effectively decoupling the electric field across neighbouring regions of the diode. As a result, the implanted section behaves as a high-resistance barrier, screening the applied bias and preventing carrier transport.

To quantify the change in device performance resulting from ion implantation, we first model the effect of implantation on the optical properties of the material. Based on Stopping and Range of Ions in Matter (SRIM) simulations, we confirm that the process operates well below the amorphization threshold; this means that defect states are formed in the valence band without generating a continuous amorphous GaAsO_*x*_ network that would lead to large variations in the complex refractive index $\tilde{n}$. Consequently, we estimate the change in $\tilde{n}$ from the corresponding reduction in charge mobility using a Drude model. This yields an approximate refractive index change of *Δn* ~ 4 × 10^−3^ and *Δk* ~ 7 × 10^−5^, with further details discussed in Supplementary Note 1. These changes in refractive index are then incorporated into a finite-difference time-domain (FDTD) simulation of the device, and loss is calculated.

Figure 1c presents the calculated photonic band structure of the GPWG, with the shaded yellow region indicating the guided mode of interest used in FDTD simulations. The transmission characteristics of this mode under various perturbation parameters are summarized in Fig. 1d and 1e. These plots show the simulated average transmission loss as a function of the predicted refractive index change (*Δn*) and extinction coefficient (*Δk*) for implantation widths of 100 nm and 500 nm, respectively.

For small implantation widths of 100 nm and 500 nm, simulations reveal that the optical mode remains largely unaffected in the range we estimate to be operating in, with perturbations of ∣*Δn*∣ ~ 4 × 10^−3^ and ∣*Δk*∣ ~ 7 × 10^−5^ resulting in ≪ 1% additional loss. In contrast, the gray plane in Fig. 1d represents the expected transmission loss of an equivalent 100 nm physical etch, exceeding 30%. This comparison highlights the strong detrimental impact of physical etching on mode confinement and scattering. Moreover, at greater implantation widths of 500 nm Fig. 1e, the guided mode continues to exhibit minimal sensitivity to the implanted region, showing only a marginal increase in transmission loss compared with the 100 nm case of ~0.04%. The corresponding black region again represents a large optical loss associated with physical etching of the same width, which exceeds 50%.

### Electrical characteristics

**Fig. 2:**
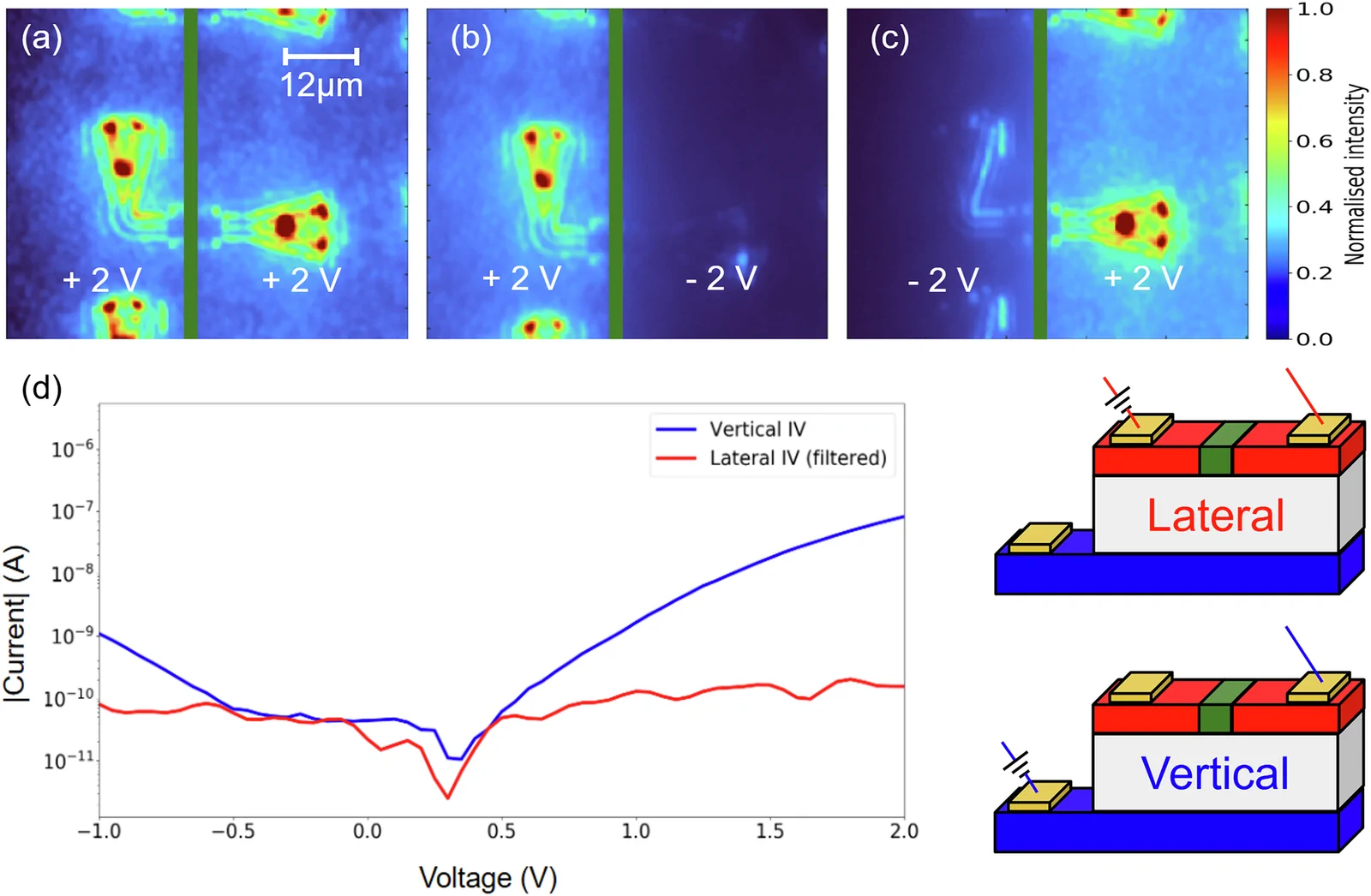
Electrical characteristics of the implanted diode. **a**–**c** PL images showing emission from two regions of a diode under different applied biases. The green pseudo-colour strip marks the implanted region. **a** Both left and right sections are forward biased at +2V, resulting in emission across the full device. **b**, **c** Decoupling of adjacent diode sections is demonstrated by locally forward biasing one section (+2V) while reverse biasing the neighbouring section (−2V), resulting in selective quenching of emission in the reverse-biased region. **d** Measured IV characteristics comparing vertical transport across the membrane (blue line) and lateral transport across the implanted barrier (red line), demonstrating strong suppression of lateral current from the implantation.

In order to assess the effectiveness of the implanted section, we perform bias-dependent PL imaging and measure the electrical characteristics of the device. Figure 2a–c shows PL images of an implanted device under different electrical bias configurations. When one side of the diode is held under reverse bias (−2V), QD emission is strongly quenched due to enhanced carrier tunnelling, resulting in the localized dark regions seen in Fig. 2b, c. The ability to quench from either side of the device separately confirms some lateral electrical isolation.

The electrical properties of the implantation strip are further demonstrated through IV measurements. Figure 2d illustrates the two measurement geometries used: (i) vertical, where current flows through the membrane, and (ii) lateral, where current flow is measured across the implanted region. The vertical measurement exhibits typical *p-i-n* diode behaviour, with an exponential increase in current above a turn-on voltage of 1.5 V. In contrast, the lateral measurement across the implanted section shows a strongly suppressed current, remaining orders of magnitude lower than the vertical current flow at similar voltage. At reverse biases beyond −2 V, we observe breakdown behaviour in the lateral diode. From the slope of the steepest linear segment of the lateral trace in Fig. 2d, and using the known geometry of the diode, we estimate a lower bound on the effective resistivity of the implanted region to be approximately 1.4 × 10^5^ *Ω* ⋅ m, a similar value shown in previous studies^43^. This corresponds to an increase in resistivity of around eight orders of magnitude compared to the unimplanted membrane, confirming that ion implantation provides some electrical isolation without the need for physical etching (baseline *p-i-n* resistivity is discussed further in Supplementary Note 2 and additional analysis of the IV curves is discussed in Supplementary Note 3).

### Relative QD tuning

**Fig. 3:**
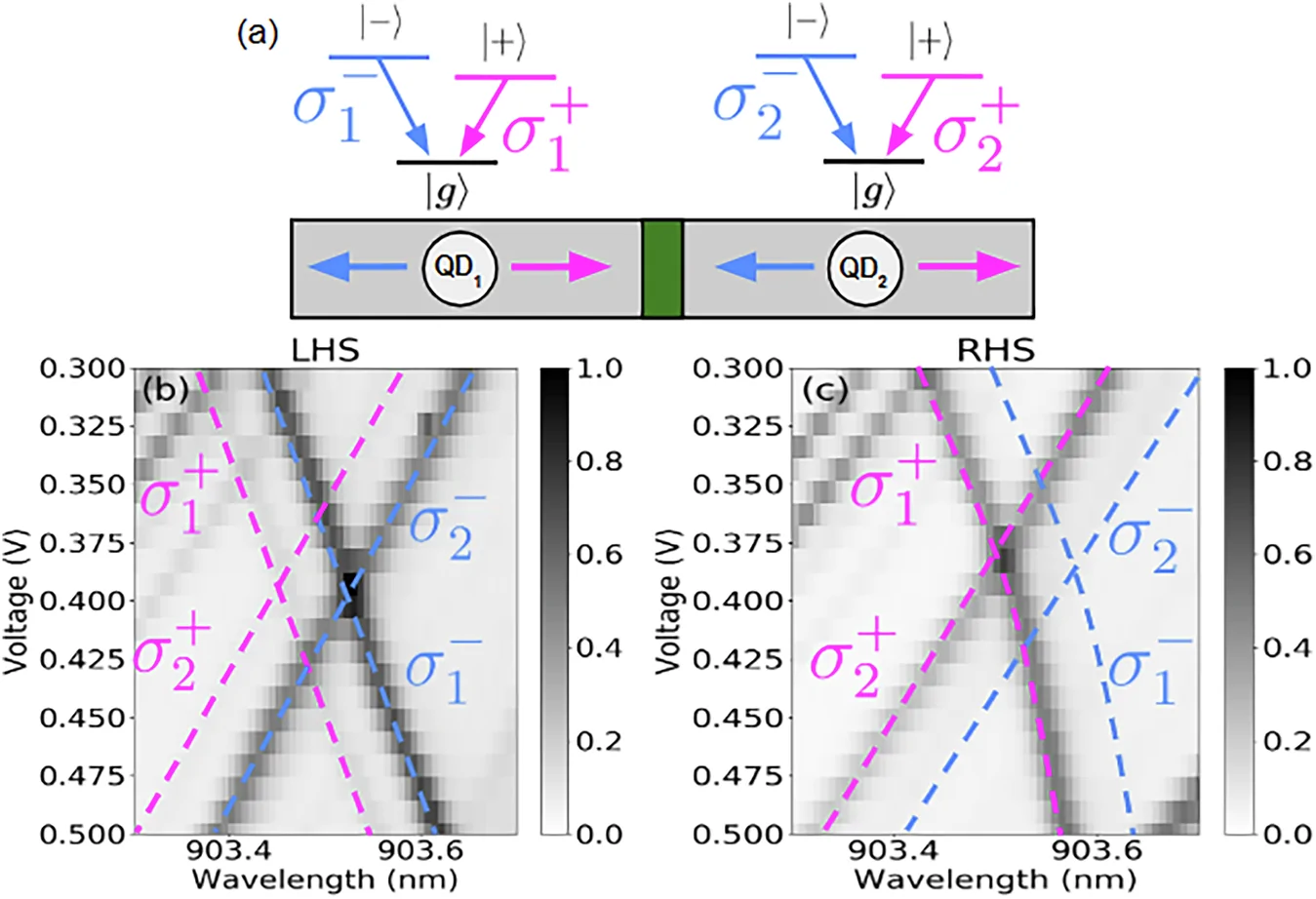
Electrical tuning of chirally coupled QDs. **a** Schematic of the chiral PhC waveguide showing two spatially separated QDs, QD_1_ and QD_2_, located on opposite sides of the implanted barrier. The subscripts 1 and 2 label the two distinct emitters. For both QD_1_ and QD_2_, the *σ*^+^ and *σ*^−^ spin transitions couple into opposite propagation directions with the same sign of chirality due to spin-momentum locking. PL maps acquired from the (**b**) left-propagating (LHS) and (**c**) right-propagating (RHS) modes, showing Stark tuning of the Zeeman-split transitions under applied voltage. Blue dashed lines trace the *σ*^−^ transitions, and magenta dashed lines trace the *σ*^+^ transitions.

Following the electrical characterisation, we perform PL measurements on two chirally coupled QDs embedded in a GPWG to demonstrate control of the detuning between separate chirally-coupled QDs. These emitters exhibit the charged exciton states ${X}_{QD1}^{-}$ and ${X}_{QD2}^{-}$. These lines were selected based on their high directionality, with the two states able to be fully tuned across one another, as illustrated in Fig. 3b, c. We apply a 0.5 T magnetic field in the Faraday geometry, which lifts the spin degeneracy of excitonic states of QDs in the waveguide, giving rise to circularly polarized transitions (*σ*^+^ and *σ*^−^) that directionally couple into opposite propagation directions of the waveguide mode, as illustrated in Fig. 3a.

The resulting PL maps are shown in Fig. 3b, c, where emission is collected via shallow-etch grating couplers from either the (b) left-hand side (LHS) or right-hand side (RHS) of the device. The *σ*^−^ states of both QDs couple preferentially to the left-propagating mode, while the *σ*^+^ couple to the right-propagating mode. The bias applied to *QD*_2_ is held constant at 1.43 V, while the bias applied *QD*_1_ is varied. Each Zeeman-split transition exhibits clear and continuous Stark tuning. Notably, no abrupt spectral jumps or drift are observed over the full tuning range, attesting to the high stability of the implanted waveguide environment. This level of spectral control is essential for the realization of indistinguishable photon sources in scalable quantum photonic circuits. We note that we observe signs of cross-talk between the two states, as evident in the PL maps. This behaviour is attributed to the physical proximity of the QDs to the diode interface (~3 and 4 *μm* for *QD*_1_ and *QD*_2_, respectively). Despite this, the data confirms that ion implantation provides sufficient decoupling of the diodes' electric fields to enable differential tuning of closely spaced emitters within the same photonic device. Where a system has differential tuning, independent tuning can be achieved through the application of ’virtual gates’^44,45^. Reduced cross-talk for emitters located further from the implantation region is shown in the Supplementary Note 4.

Importantly, both emitters are strongly directional, and we do not observe a substantial decrease in the measured chiral contrast as the emission propagates across the implanted region from either end of the waveguide, indicating that loss through the implanted region is minimal compared to physical etching, which in ideal simulations is substantial. Chiral contrast (*C*) can be defined as the contrast of the power (*P*_*L*/*R*_) emitted to the left and right propagating modes of the waveguide such that:

1 $$C=\frac{{P}_{R}-{P}_{L}}{{P}_{R}+{P}_{L}}$$

${X}_{QD1}^{-}$ exhibits a chiral contrast of 84%, and ${X}_{QD2}^{-}$ a contrast of 74%. There is only a minor asymmetry in the contrast when measured from each direction. 2% for ${X}_{QD1}^{-}$ and 13% for ${X}_{QD2}^{-}$, asymmetries that are comparable to those commonly observed in unimplanted devices^17,34,41,46,47^. This asymmetry could be attributed to weak residual backscattering at the adiabatic mode converter interface or subtle fabrication imperfections/disorder, rather than any intrinsic modification of the underlying photonic mode structure. Both emitters exhibit contrasts that would not be seen in a physically etched glide-plane waveguides, where FDTD simulations predict a pronounced reduction in chiral contrast from ~98% for an unetched device to ~38% for etch widths of 500 nm. The absence of such degradation in the implanted structures demonstrates that low-dose oxygen implantation modifies the refractive index only perturbatively, without disrupting the guided mode symmetry or the deterministic spin-photon transfer essential for chiral quantum interfacing.

**Fig. 4:**
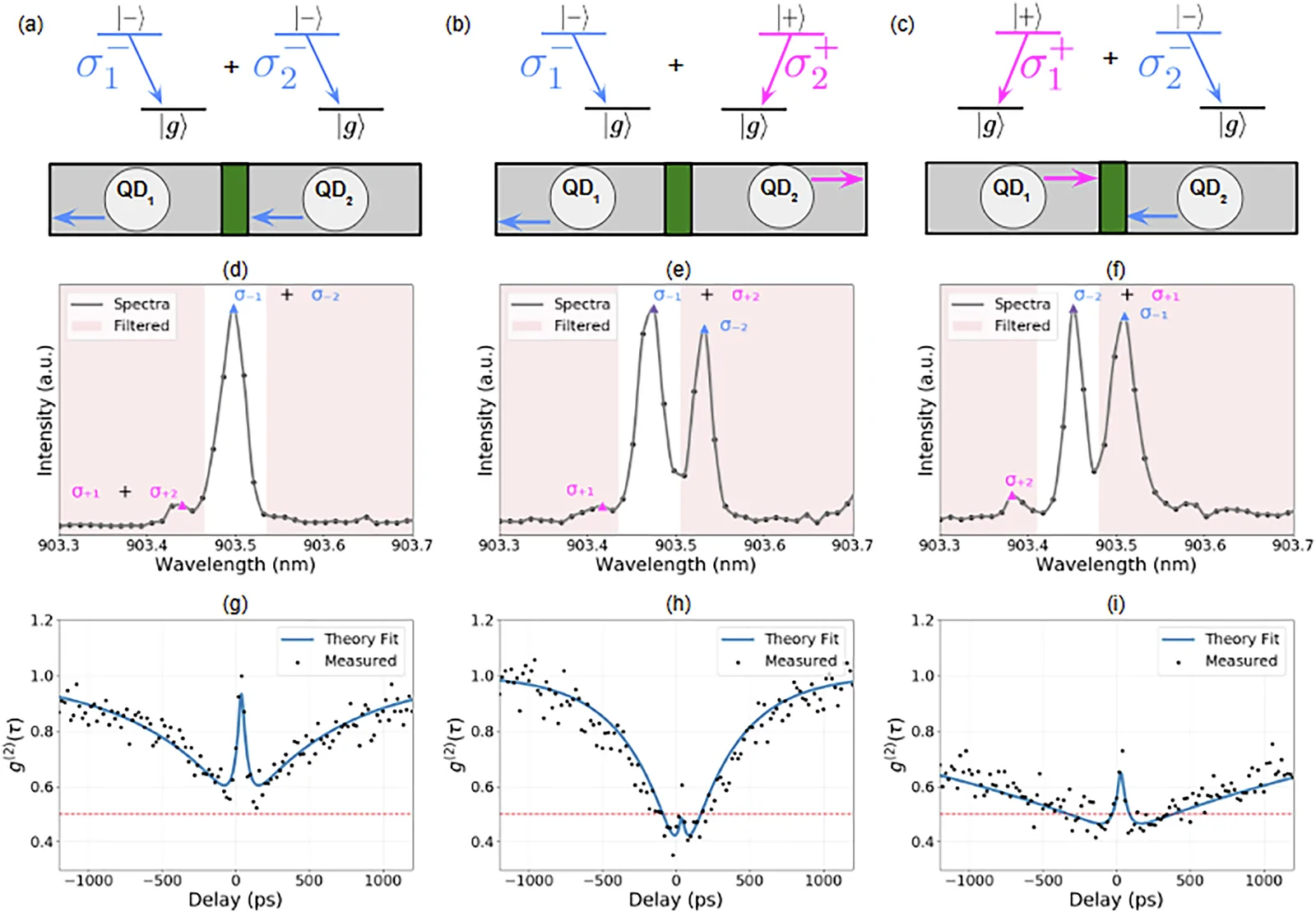
Spin-dependent photon correlations in an implanted waveguide. Spin configurations for three two-emitter correlation measurements **a** the *σ*^−^ states of ${X}_{QD1}^{-}$ and ${X}_{QD2}^{-}$ are measured, **b** the *σ*^−^ state of ${X}_{QD1}^{-}$ and *σ*^+^ of ${X}_{QD2}^{-}$ are measured, **c** the *σ*^+^ state of ${X}_{QD1}^{-}$ and *σ*^−^ of ${X}_{QD2}^{-}$ are measured PL spectra collected from the left-hand side outcoupler demonstrating the tuning of various spin pairs into resonance. (**d**) ${\sigma }_{1}^{-}$ + ${\sigma }_{2}^{-}$, (**e**) ${\sigma }_{1}^{-}$ + ${\sigma }_{2}^{+}$, (**f**) ${\sigma }_{1}^{+}$ + ${\sigma }_{2}^{-}$, with red-shaded regions indicating the spectral windows used for filtering prior to correlation measurements. **g**–**i** Second-order photon correlation functions collected from the left-hand side *g*^(2)^(*τ*) for the spin state combinations in (**a**–**c**), showing theoretical fits (solid lines) and experimental data (black dots). The quantum limit, *g*^(2)^(*τ*) = 0.5, is indicated by the red dashed line.

Having established the tuning behaviour of the two chirally-coupled QDs in our device, we proceed to investigate co-operative effects between the different spin states of the two excitons. Collective emission effects are expected only when both states couple predominantly to the same propagation direction of the guided mode. To observe this, we perform second-order photon correlation measurements on emission from pairs of the QD states, driving both QDs and collecting emission from the left outcoupler, as in Fig. 3a. For each measurement, a different pair of the QD transitions is tuned onto resonance, and a 70pm band-pass filter is used to isolate emission from the resonant pair. The three spin-pair configurations studied are shown in Fig. 4a–f: (a, d) ${\sigma }_{1}^{-}$ + ${\sigma }_{2}^{-}$, (b, e) ${\sigma }_{1}^{-}$ + ${\sigma }_{2}^{+}$, and (c, f) ${\sigma }_{1}^{+}$ + ${\sigma }_{2}^{-}$ for ${X}_{QD1}^{-}$ and ${X}_{QD2}^{-}$. The corresponding autocorrelation functions *g*^(2)^(*τ*) are plotted in Fig. 4g–i.

In configuration Fig. 4g the two spin states are coupled to the same propagating direction, and as a result, strong photon bunching is observed at zero time delay, indicating interference between the photons emitted into the waveguide mode. In contrast, for configurations Fig. 4h and Fig. 4i the two spin states measured are coupled predominantly to opposing propagating directions. These measurements show dramatically reduced or absent bunching, reflecting the reduced coupling between states that are coupled to opposing waveguide modes. The three HBT measurements have been fitted to a theoretical model, using a consistent set of parameters across the measurements. We note that the differing behaviour seen in Fig. 4h and Fig. 4i may be caused by the different optical properties of the two QDs, predominantly the different decay rates and chiral contrasts. The modelling and properties of the QDs are discussed further in Supplementary Note 5.

In addition to demonstrating the chiral behaviour of this pair of emitters, this technique provides a sensitive probe of the long-term spectral stability of the QDs. Spectrally indistinguishable photons interfere to produce clear bunching or antibunching signatures only when their emission energies remain stable within the photon coherence time^48^. Substantial spectral diffusion or Stark jitter would wash out these correlations, leading to a degraded visibility. Thus, the observation of robust spin-dependent photon correlations demonstrates that ion implantation maintains the expected spectral stability of the emitters over extended measurement times.

## Discussion

We demonstrate a scalable method for achieving electrical tuning of excitons in spatially separated QDs embedded in a photonic crystal waveguide. The implantation of targeted ions into the *p*-doped region of a *p-i-n* diode creates a high-resistivity barrier that enables Stark tuning while preserving the optical properties of the surrounding nanophotonic structure. Unlike physical etching, which introduces detrimental scattering and degrades photonic mode integrity, our approach maintains the high-quality spin-photon interfaces essential for scalable chiral quantum networks.

PL imaging and I-V measurements confirm the decoupling of diode sections when applying voltage to adjacent sections separated by implantation, while correlation experiments demonstrate long-term spectral stability and spin-resolved addressability of the QDs. In particular, spin-resolved second-order correlation *g*^(2)^(*τ*) measurements reveal direction-dependent photon bunching with negligible Stark-induced spectral jitter. This stability confirms that our implantation scheme supports extended experimental operation, a crucial requirement for protocols involving long measurement times, high-visibility interference, and quantum network synchronization^49,50^.

These results confirm selective spin-photon coupling, robust spectral stability, and coherent interference between remote emitters—key ingredients for scalable chiral quantum networks. In chiral quantum systems, where spin-momentum locking routes photons of opposite helicity into distinct propagation channels, interference between indistinguishable emitters enables deterministic entanglement generation, entanglement swapping, and quantum repeater functionality^51^. Demonstrating stable and selective spin-photon correlations therefore establishes spin-photon interfacing between indistinguishable emitters mediated by the waveguide, representing a critical milestone toward functional multi-emitter quantum networks^52^. More broadly, the demonstrated stability and addressability highlight the suitability of our non-destructive tuning method for scalable architectures that rely on multi-emitter interference in chiral photonic circuits, including deterministic entanglement generation^9^, quantum repeaters^53^, and chiral quantum optical interfaces.

Importantly, the implantation-based scheme is not restricted to the specific platform studied here. These methods can be adapted to other III-V systems, such as InP membrane photonic crystal devices^54,55^ incorporating InAs/InP QDs^54,56–59^, where telecom C-band operation and waveguide-based chiral interfaces could be realized with independent electrical control of multiple emitters, enabled by recently demonstrated Stark tuning in this material system^60^.

Our work therefore highlights ion implantation as a potentially powerful tool for independently addressing and tuning multiple emitters on the same chip, fully compatible with photonic integrated circuit architectures. The development of such established methods thus represents a key step toward scalable multi-qubit operations and integrated quantum photonic circuits.

## Methods

### Fabrication

The sample used in this work consists of a 170 nm GaAs membrane grown by molecular beam epitaxy (MBE) on top of a 1.1 *μ* m thick Al_0.6_Ga_0.4_As sacrificial layer, supported by a GaAs substrate (wafer structure shown in Fig. 1(a)). The membrane is configured as a *p-i-n* diode heterostructure, with a single layer of InAs QDs embedded at its center. The top 30 nm *p*-layer of the membrane is doped with carbon at a concentration of 2 × 10^19^cm^−3^, while the bottom 30 nm is *n*-doped with silicon at a concentration of 2 × 10^18^cm^−3^. In addition, there are two Al_0.3_Ga_0.7_As barriers on either side of the QD layer. The wider band gap of AlGaAs suppresses carrier tunnelling and leakage currents, allowing single QD Stark tuning of a few meV^61^.

Prior to fabrication of any photonic structures, a 600nm thick CSAR soft mask is deposited and patterned using electron beam lithography (EBL). The exposed areas of the sample are then bombarded with O^−^ ions at a dose of 2 × 10^12^cm^2^ accelerated to 40 keV, forming 500 nm tracks across diode sections (The choice of width is justified in Supplementary Note 6). The implantation is performed in a 7/22 orientation (tilt and azimuthal angle for in-plane rotation) to avoid ion channeling caused by alignment with the major crystal axis. The PhC is then patterned using a SiO_2_ hard mask with EBL. The patterns are transferred into the GaAs membrane using inductively coupled plasma reactive ion etching (ICP-RIE). Electrical contacts to the *p*- and *n*-doped layers are made using Ti/Au metallization. This configuration allows for electrical biasing of the laterally separated regions of the diode by placing contact pads, which can be wire-bonded after full device fabrication and connected to a ceramic chip carrier. Following this, the wafer is placed in hydrofluoric acid (HF) to selectively remove the sacrificial layer of Al_0.6_Ga_0.4_As, releasing the membrane and forming suspended photonic structures. To avoid mechanical damage during drying in HF, a critical point drying process is employed.

## Supplementary information

Supplementary Information

## Data Availability

The datasets generated and/or analyzed during the current study are not publicly available because they form part of ongoing related research projects and collaborative development activities with other institutions, and include fabrication-sensitive information, but are available from the corresponding author on reasonable request.

## References

[1] Mahmoodian, S., Lodahl, P. & Sørensen, A. S. Quantum networks with chiral-light-matter interaction in waveguides. *Phys. Rev. Lett.* **117**, 240501, 10.1103/PhysRevLett.117.240501 (2016). 28009207

[2] Pichler, H., Ramos, T., Daley, A. J. & Zoller, P. Quantum optics of chiral spin networks. *Phys. Rev. A* **91**, 042116, 10.1103/PhysRevA.91.042116 (2015).

[3] Madsen, L. S. et al. Quantum computational advantage with a programmable photonic processor. *Nature* **606**, 75–81, 10.1038/s41586-022-04725-x (2022). 35650354 PMC9159949

[4] Yang, Y. et al. Programmable quantum circuits in a large-scale photonic waveguide array. *npj Quantum Inf.* **11**, 19. 10.1038/s41534-024-00934-6 (2025).

[5] Slussarenko, S. & Pryde, G. J. Photonic quantum information processing: A concise review. *Appl. Phys. Rev.* **6**, 041303, 10.1063/1.5115814 (2019).

[6] Niu, D., Zhang, Y., Shabani, A. & Shapourian, H. All-photonic one-way quantum repeaters with measurement-based error correction. *npj Quantum Inf.* **9**, 106. 10.1038/s41534-023-00775-9 (2023).

[7] Duda, M., Martin, N. J., Mills, E. O., Wilson, L. R. & Kok, P. Routing single photons with quantum emitters coupled to nanostructures. *Appl. Phys. Rev.* **13**, 021323, 10.1063/5.0264750 (2026).

[8] Tomm, N. et al. A bright and fast source of coherent single photons. *Nat. Nanotechnol.* **16**, 399–403, 10.1038/s41565-020-00831-x (2021). 33510454

[9] Somaschi, N. et al. Near-optimal single-photon sources in the solid state. *Nat. Photonics* **10**, 340–345, 10.1038/nphoton.2016.23 (2016).

[10] Meng, Y. et al. Deterministic photon source of genuine three-qubit entanglement. *Nat. Commun.* **15**, 7774. 10.1038/s41467-024-52086-y (2024). 39237490 PMC11377771

[11] Arcari, M. et al. Near-unity coupling efficiency of a quantum emitter to a photonic crystal waveguide. *Phys. Rev. Lett.* **113**, 093603, 10.1103/PhysRevLett.113.093603 (2014). 25215983

[12] Lodahl, P., Mahmoodian, S. & Stobbe, S. Interfacing single photons and single quantum dots with photonic nanostructures. *Rev. Mod. Phys.* **87**, 347–400, 10.1103/RevModPhys.87.347 (2015).

[13] Quan, Q. & Loncar, M. Deterministic design of wavelength-scale, ultra-high-Q photonic crystal nanobeam cavities. *Opt. Express* **19**, 18529–18542, 10.1364/OE.19.018529 (2011). 21935223

[14] Germanis, S. et al. Waveguide excitation and spin pumping of chirally coupled quantum dots. *Optica* **12**, 1689–1696, 10.1364/OPTICA.569882 (2025).

[15] Siampour, H. et al. Observation of large spontaneous emission rate enhancement of quantum dots in a broken-symmetry slow-light waveguide. *npj Quantum Inf.* **9**, 15. 10.1038/s41534-023-00686-9 (2023).

[16] Söllner, I. et al. Deterministic photon-emitter coupling in chiral photonic circuits. *Nat. Nanotechnol.* **10**, 775–778, 10.1038/nnano.2015.159 (2015). 26214251

[17] Martin, N. J. et al. Topological and conventional nanophotonic waveguides for directional integrated quantum optics. *Phys. Rev. Res.* **6**, L022065, 10.1103/PhysRevResearch.6.L022065 (2024).

[18] Hauff, N. V., Le Jeannic, H., Lodahl, P., Hughes, S. & Rotenberg, N. Chiral quantum optics in broken-symmetry and topological photonic crystal waveguides. *Phys. Rev. Res.* **4**, 023082, 10.1103/PhysRevResearch.4.023082 (2022).

[19] Rastelli, A., Kiravittaya, S. & Schmidt, O. G.*Growth and control of optically active quantum dots* (Springer Berlin Heidelberg, 2009).

[20] Zhai, L. et al. Low-noise GaAs quantum dots for quantum photonics. *Nat. Commun.* **11**, 4745. 10.1038/s41467-020-18625-z (2020). 32958795 PMC7506537

[21] Lodahl, P. et al. Chiral quantum optics. *Nature* **541**, 473–480, 10.1038/nature21037 (2017). 28128249

[22] Staunstrup, M. J. R. et al. Direct observation of a few-photon phase shift induced by a single quantum emitter in a waveguide. *Nat. Commun.* **15**, 7583. 10.1038/s41467-024-51805-9 (2024). 39217156 PMC11365957

[23] Hallett, D. et al. Electrical control of nonlinear quantum optics in a nano-photonic waveguide. *Optica* **5**, 644–650, 10.1364/OPTICA.5.000644 (2018).

[24] Hallacy, L. et al. Nonlinear quantum optics at a topological interface enabled by defect engineering. *npj Nanophoton.* **2**, 9, 10.1038/s44310-025-00057-6 (2025).

[25] Yang, F., Lund, M. M., Pohl, T., Lodahl, P. & Mølmer, K. Deterministic photon sorting in waveguide QED systems. *Phys. Rev. Lett.* **128**, 213603, 10.1103/PhysRevLett.128.213603 (2022). 35687472

[26] Gonzalez-Ballestero, C., Gonzalez-Tudela, A., Garcia-Vidal, F. J. & Moreno, E. Chiral route to spontaneous entanglement generation. *Phys. Rev. B* **92**, 155304, 10.1103/PhysRevB.92.155304 (2015).

[27] Mirza, I. M., Hoskins, J. G. & Schotland, J. C. Chirality, band structure, and localization in waveguide quantum electrodynamics. *Phys. Rev. A* **96**, 053804, 10.1103/PhysRevA.96.053804 (2017).

[28] Mirza, I. M., Hoskins, J. G. & Schotland, J. C. Dimer chains in waveguide quantum electrodynamics. *Opt. Commun.* **463**, 125427, 10.1016/j.optcom.2020.125427 (2020).

[29] Ramos, T., Pichler, H., Daley, A. J. & Zoller, P. Quantum spin dimers from chiral dissipation in cold-atom chains. *Phys. Rev. Lett.* **113**, 237203, 10.1103/PhysRevLett.113.237203 (2014). 25526153

[30] Li, T., Miranowicz, A., Hu, X., Xia, K. & Nori, F. Quantum memory and gates using a Λ-type quantum emitter coupled to a chiral waveguide. *Phys. Rev. A* **97**, 062318, 10.1103/PhysRevA.97.062318 (2018).

[31] Schrinski, B., Lamaison, M. & Sørensen, A. S. Passive quantum phase gate for photons based on three level emitters. *Phys. Rev. Lett.* **129**, 130502, 10.1103/PhysRevLett.129.130502 (2022). 36206425

[32] Levy-Yeyati, T., Vega, C., Ramos, T. & González-Tudela, A. Passive photonic CZ gate with two-level emitters in chiral multimode waveguide QED. *PRX Quantum* **6**, 010342, 10.1103/PRXQuantum.6.010342 (2025).

[33] Hallett, D., Foster, A. P., Whittaker, D., Skolnick, M. S. & Wilson, L. R. Engineering chiral light-matter interactions in a waveguide-coupled nanocavity. *ACS Photonics* **9**, 706–713, 10.1021/acsphotonics.1c01806 (2022). 35434181 PMC9007567

[34] Martin, N. J. et al. Purcell-enhanced, directional light-matter interaction in a waveguide-coupled nanocavity. *Optica* **12**, 1100–1108, 10.1364/OPTICA.561630 (2025).

[35] Shapourian, H. & Shabani, A. Modular architectures to deterministically generate graph states. *Quantum* **7**, 935, 10.22331/q-2023-03-02-935 (2023).

[36] Chu, X.-L. et al. Independent electrical control of two quantum dots coupled through a photonic-crystal waveguide. *Phys. Rev. Lett.* **131**, 033606, 10.1103/PhysRevLett.131.033606 (2023). 37540854

[37] Hallett, D. et al. Controlling coherence between waveguide-coupled quantum dots. *Phys. Rev. Appl.* **25**, L021003, 10.1103/1xsb-bf5r (2026).

[38] Patterson, M. et al. Disorder-induced incoherent scattering losses in photonic crystal waveguides: Bloch mode reshaping, multiple scattering, and breakdown of the Beer-Lambert law. *Phys. Rev. B* **80**, 195305, 10.1103/PhysRevB.80.195305 (2009).

[39] Thon, S. M. et al. Independent electrical tuning of separated quantum dots in coupled photonic crystal cavities. *Appl. Phys. Lett.* **99**, 161102, 10.1063/1.3651491 (2011).

[40] Yu, X. et al. Ge ion-implanted photonic devices and annealing for emerging applications. *Micromachines* **13**, 10.3390/mi13020291 (2022). PMC8880043 35208415

[41] Coles, R. J. et al. Chirality of nanophotonic waveguide with embedded quantum emitter for unidirectional spin transfer. *Nat. Commun.* **7**, 11183. 10.1038/ncomms11183 (2016). 27029961 PMC4821884

[42] Yin, R. et al. Physical mechanism of field modulation effects in ion-implanted edge termination of vertical GaN Schottky barrier diodes. *Fundam. Res.* **2**, 629–634, 10.1016/j.fmre.2021.11.027 (2022). 38934000 PMC11197601

[43] Pearton, S. Ion implantation doping and isolation of iii-v semiconductors. *Nucl. Instrum. Methods Phys. Res. B: Beam Interact. Mater.* **59-60**, 970–977, 10.1016/0168-583X(91)95744-X (1991).

[44] Volk, C. et al. Loading a quantum-dot-based “qubyte” register. *npj Quantum Inf.* **5**, 29. 10.1038/s41534-019-0146-y (2019).

[45] Hsiao, T.-K. et al. Efficient orthogonal control of tunnel couplings in a quantum dot array. *Phys. Rev. Appl.* **13**, 054018, 10.1103/PhysRevApplied.13.054018 (2020).

[46] Mehrabad, M. J. et al. Chiral topological photonics with an embedded quantum emitter. *Optica* **7**, 1690–1696, 10.1364/OPTICA.393035 (2020).

[47] Mehrabad, M. J. et al. Chiral topological add–drop filter for integrated quantum photonic circuits. *Optica* **10**, 415–421, 10.1364/OPTICA.481684 (2023).

[48] Santori, C., Fattal, D., Vučković, J., Solomon, G. S. & Yamamoto, Y. Indistinguishable photons from a single-photon device. *Nature* **419**, 594–597, 10.1038/nature01086 (2002). 12374958

[49] Senellart, P., Solomon, G. & White, A. High-performance semiconductor quantum-dot single-photon sources. *Nat. Nanotechnol.* **12**, 1026–1039, 10.1038/nnano.2017.218 (2017). 29109549

[50] Warburton, R. J. Single spins in self-assembled quantum dots. *Nat. Mater.* **12**, 483–493, 10.1038/nmat3585 (2013). 23695745

[51] Suárez-Forero, D., Jalali Mehrabad, M., Vega, C., González-Tudela, A. & Hafezi, M. Chiral quantum optics: Recent developments and future directions. *PRX Quantum* **6**, 020101, 10.1103/PRXQuantum.6.020101 (2025).

[52] Ramos, T., Vermersch, B., Hauke, P., Pichler, H. & Zoller, P. Non-Markovian dynamics in chiral quantum networks with spins and photons. *Phys. Rev. A* **93**, 062104, 10.1103/PhysRevA.93.062104 (2016).

[53] Vajner, D. A., Rickert, L., Gao, T., Kaymazlar, K. & Heindel, T. Quantum communication using semiconductor quantum dots. *Adv. Quant. Technol.* **5**, 2100116 (2022).

[54] Phillips, C. L. et al. Purcell-enhanced single photons at telecom wavelengths from a quantum dot in a photonic crystal cavity. *Sci. Rep.* **14**, 4450. 10.1038/s41598-024-55024-6 (2024). 38396018 PMC11310300

[55] Ma, C. et al. Circular photonic crystal grating design for charge-tunable quantum light sources in the telecom C-band. *Opt. Express* **32**, 14789–14800, 10.1364/OE.517758 (2024). 38859415

[56] Ge, Z., Chung, T., He, Y.-M., Benyoucef, M. & Huo, Y. Polarized and bright telecom C-band single-photon source from InP-based quantum dots coupled to elliptical Bragg gratings. *Nano Lett.* **24**, 1746–1752 (2024). 38286024 10.1021/acs.nanolett.3c04618

[57] Holewa, P. et al. High-throughput quantum photonic devices emitting indistinguishable photons in the telecom C-band. *Nat. Commun.* **15**, 3358 (2024). 38637520 10.1038/s41467-024-47551-7 PMC11026509

[58] Sala, E. M., Godsland, M., Na, Y. I., Trapalis, A. & Heffernan, J. Droplet epitaxy of InAs/InP quantum dots via MOVPE by using an InGaAs interlayer. *Nanotechnology* **33**, 065601 (2022). 10.1088/1361-6528/ac3617 34731846

[59] Sala, E. M., Na, Y. I., Godsland, M. & Heffernan, J. Self-assembled InAs quantum dots on InGaAsP/InP (100) by modified droplet epitaxy in metal–organic vapor phase epitaxy around the Telecom C-band for quantum photonic applications. *Phys. Status Solidi RRL* **18**, 2300340 (2024).

[60] Martin, N. J. et al. Stark tuning and charge state control in individual telecom c-band quantum dots. *Appl. Phys. Lett.* **127**, 194001, 10.1063/5.0302840 (2025).

[61] Bennett, A. J. et al. Giant Stark effect in the emission of single semiconductor quantum dots. *Appl. Phys. Lett.* **97**, 031104, 10.1063/1.3460912 (2010).

