# Supplementary material for: Chiral quantum optics with scalable quantum dot dimers
Source: Npj Nanophoton. 2026 Sep 9;3(1):40. doi: 10.1038/s44310-026-00148-y (PMC13558063; doi:10.1038/s44310-026-00148-y)
Supplement: Supplementary file 1 [file 44310_2026_148_MOESM1_ESM.pdf]

Supplementary Information: Chiral Quantum Optics with Scalable Quantum Dot Dimers

L. Hallacy,[1,*] D. Hallett,[1] A. Fenzl,[1] N.J. Martin,[1] R. Dost,[1] A.Verma,[2] J. Fletcher,[3] I. Farrer,[2] L. Antwis,[3] M.S. Skolnick,[1] and L.R. Wilson[1]

[1] *School of Mathematical and Physical Sciences, University of Sheffield, Sheffield, S3 7RH, UK*

[2] *School of Electrical and Electronic Engineering, University of Sheffield, Sheffield, S1 3JD, UK*

[3] *Ion Beam Centre, University of Surrey, Guildford, UK, GU2 7XH*

(Dated: July 28, 2026)

Contents:

Supplementary Note 1: Effects of implantation on optical properties

Supplementary Note 2: Resistivity of p-layer

Supplementary Note 3: Further investigation of current between diodes

Supplementary Note 4: Cross-talk free tuning of QDs

Supplementary Note 5: Theory of two-emitter second-order correlations

Supplementary Note 6: Bulk diode tests

* lmhallacy1@sheffield.ac.uk

SUPPLEMENTARY NOTE 1: EFFECTS OF IMPLANTATION ON OPTICAL PROPERTIES

To evaluate the structural effects of oxygen implantation, we used Stopping and Range of Ions in Matter (SRIM) simulations to estimate the amorphization limit for GaAs under 40 keV O^+ bombardment. From the simulated damage profiles, the critical fluence for amorphization at 40 keV is $\Phi_{\mathrm{amorph}} \approx 3\times10^{15}\,\mathrm{cm}^{-2}$. Our implantation fluence of $2\times10^{12}\,\mathrm{cm}^{-2}$ is therefore more than three orders of magnitude lower, yielding a damage fraction:

$$r = \frac{\Phi}{\Phi_{\mathrm{amorph}}} \approx 7 \times 10^{-4}. \tag{S1}$$

This places the process well within the sub-threshold crystalline regime, where only isolated point defects and small clusters are created without the formation of any continuous amorphous $GaAsO_x$ network [1].

Oxygen implantation introduces a high density of mid-gap defect centres, such as vacancy–oxygen complexes, anti-sites, and related deep traps that efficiently capture carriers [2]. When the trap density exceeds the shallow acceptor density, the free-hole population collapses despite the dopant concentration remaining unchanged. This "complete hole freeze-out" reflects the localization of holes in deep, non-thermally ionizable states, rendering the implanted region effectively semi-insulating.

To estimate the optical perturbation associated with this carrier removal, we use a simple Drude free-carrier model [3]. We assume complete hole freeze-out and therefore take the change in free-hole density to be:

$$\Delta N_h = -2 \times 10^{19}\ \mathrm{cm}^{-3}. \tag{S2}$$

The corresponding change in the real part of the refractive index is:

$$\Delta n = -\frac{e^2\lambda^2}{8\pi^2 c^2 \epsilon_0 n_0}\left(\frac{\Delta N_h}{m_h^*}\right), \tag{S3}$$

where $m_h^* = 0.51 m_0$, $n_0 = 3.4$, and $\lambda = 900$ nm. Substituting these values gives:

$$\Delta n \approx +4 \times 10^{-3}, \tag{S4}$$

consistent with a small reduction of the negative plasma contribution to the refractive index [3]. To obtain a simple estimate for the imaginary part, we include Drude damping through the hole scattering time:

$$\tau_h = \frac{\mu_h m_h^*}{e}, \tag{S5}$$

where μ_h is the hole mobility. Using a representative room-temperature value for heavily doped p-type GaAs of:

$$\mu_h \approx 100 \text{ cm}^2\,\text{V}^{-1}\,\text{s}^{-1}, \tag{S6}$$

which is consistent with the expected majority-hole mobility scale in heavily doped GaAs [4], we estimate the change in extinction coefficient as:

$$\Delta k \approx -\frac{\Delta n}{\omega \tau_h}, \qquad \omega = \frac{2\pi c}{\lambda}. \tag{S7}$$

This gives:

$$\Delta k \approx -6.9 \times 10^{-5}. \tag{S8}$$

The negative sign of Δk indicates that the free-carrier absorption is reduced when the holes are frozen out, as expected from the same simple Drude picture used above [3].

The corresponding complex refractive-index change may therefore be written as:

$$\Delta \tilde{n} = \Delta n + i\Delta k \approx (4 \times 10^{-3}) - i(7 \times 10^{-5}). \tag{S9}$$

SUPPLEMENTARY NOTE 2: RESISTIVITY OF P-LAYER

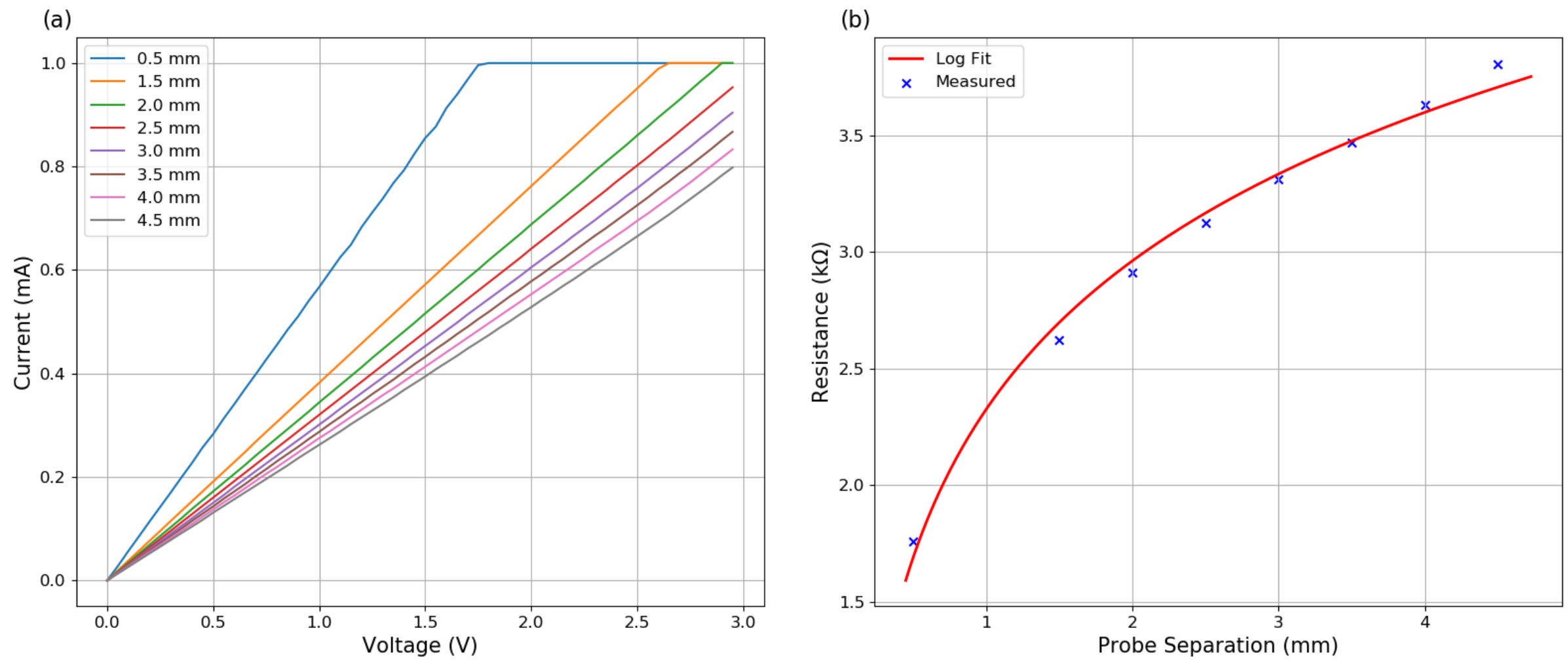

Figure S1: **Electrical characterisation of the unimplanted p-doped GaAs layer in bulk diodes.** (a) Current–voltage measurements through the p-doped layer for probe separations ranging from 0.5 mm to 4.5 mm, showing the linear low-bias regime used for resistance extraction. (b) Measured two-terminal resistance as a function of probe separation. Blue crosses show the measured resistance values, and the red line shows the logarithmic fit based on Eq.(S10).

In the paper, we calculate the resistivity of the implanted material using the resistance across the split-diode structure. To establish a baseline resistivity for the p-layer before implantation, we contact lithographically defined Ti/Au pads to the top of the wafer with varying separations d. Large separations must be used because the conductive p-layer has very low resistivity. Low-bias current–voltage measurements (Figure S1(a)) are performed for each separation. These measurements show ohmic behaviour at all separations, and the resistance extracted from the linear IV slope is plotted as a function of d.

To calculate resistivity we implement a spreading-resistance model [5] since the lateral transport is dominated by the 30 nm p-doped layer. The large aspect ratio between the probe separation (d) and the film thickness (t) is of the order 10^3 allowing an approximation of a two-dimensional current distribution, consistent with the spreading resistance formal-

ism [6]. The Au/p-GaAs contacts are sufficiently small compared to the probe spacing that current spreads radially from one pad before entering the other, reproducing the geometry considered in thin-film models. In this regime, the measured two-terminal resistance is governed by the logarithmic form of Eq.(S10), allowing extraction of an effective sheet resistivity for the p-layer defined as:

$$R(d) = \frac{\rho}{\pi t} \ln\left(\frac{d}{r_0}\right), \tag{S10}$$

where ρ is the film resistivity and r_0 is the effective contact radius, typically larger than the pad radius due to current crowding.

A nonlinear least-squares fit of Eq.(S10) to the measured data (Figure S1(b)) yielded $\rho = (0.85 \pm 0.03) \times 10^{-6}\,\Omega{\cdot}\mathrm{m}$, $r_0 = (73 \pm 10)\,\mu\mathrm{m}$, and a sheet resistance $R_s = (280 \pm 13)\,\mathrm{k}\Omega/\mathrm{cm}$, with $R^2 = 0.99$. The spreading-resistance model is valid here because the lateral transport is confined to the 30 nm p-layer and the probe spacing exceeds the layer thickness by over three orders of magnitude, ensuring a two-dimensional current distribution [6].

SUPPLEMENTARY NOTE 3: FURTHER INVESTIGATION OF CURRENT BETWEEN DIODES

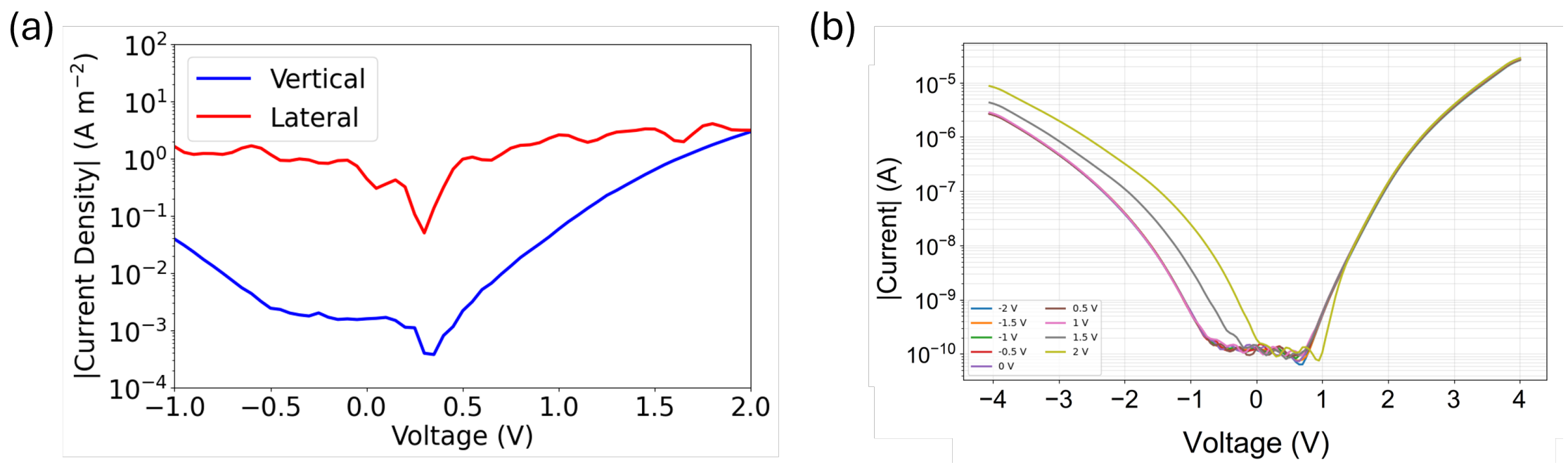

Figure S2: **Electrical response of adjacent ion implanted GaAs diodes in lateral and vertical schemes.** (a) Current density-voltage performance of the (vertical) diode, and the (lateral) implantation region (blue and red line respecitvely). (b) Current voltage properties of the vertical diode 1, for different voltages applied to diode 2.

We present some further information on the current flow across the implanted junction of the device studied in the main manuscript. Figure S2 (a) shows the current density across the implanted junction, compared to the vertial current density. This uses the same data in Figure.2(d) The vertical diode demonstrates diode-like behaviour, while the lateral current flow is nearly voltage-independent. Due to the relatively small cross-section of the implantation (approximately 1000 μm by 30 nm), the lateral current density is comparatively high.

Figure S2(b) shows how the vertical current of diode 1 is affected by the bias applied to diode 2. We see that the IV properties of diode 1 are relatively unaffected by the bias applied to the adjacent diode, only seeing a difference when a voltage $> 1.5V$ is used. For the voltage range used in this work (0.3-0.7 V), no change in current is observed.

SUPPLEMENTARY NOTE 4: CROSS-TALK FREE TUNING OF QDS

The QDs used in the main paper exhibit significant cross-talk, in which the voltage applied to one diode has an effect on the QD in the other section. This behavior is only seen for emitters in close proximity to the interface. Figure S3 shows a tuning map for a pair of emitters in the same sample. These emitters are positioned further away from the interface. The cross-talk for these emitters is greatly reduced, although a small amount of cross-talk is still present.

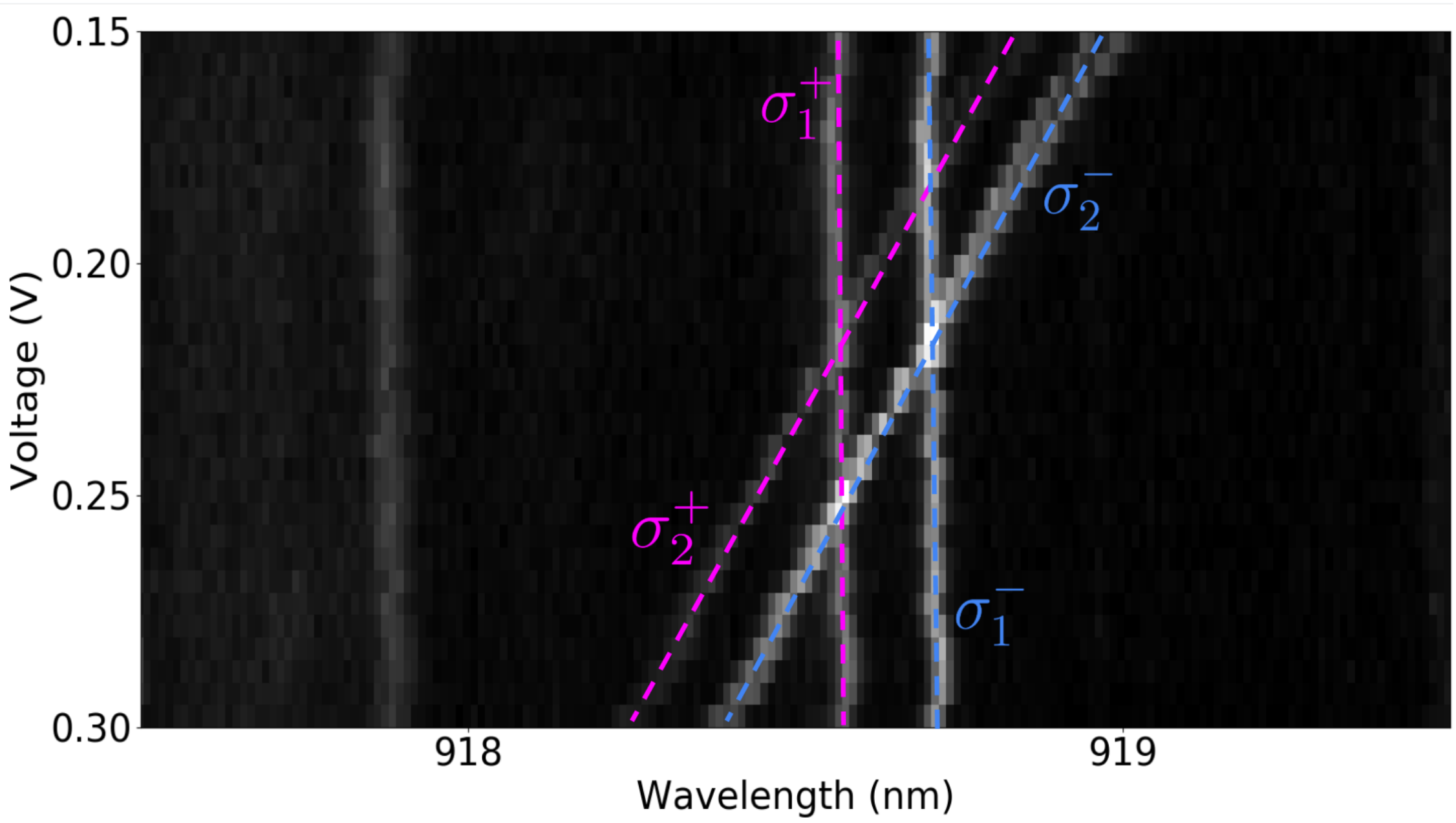

Figure S3: **Electrical tuning of chirally coupled QDs far away from implantation.** PL map recorded under the same excitation and contacting scheme as in Fig.3(a), showing the bias-dependent tuning of two QDs located in the taper region of the photonic crystal waveguide ($\gtrsim 6\mu m$ away from implantation).Blue dashed lines trace the σ^- transitions, magenta dashed lines trace the σ^+ transitions.

SUPPLEMENTARY NOTE 5: THEORY OF TWO-EMITTER SECOND-ORDER CORRELATIONS

Master equation

We consider a system of two spatially separated quantum emitters (QDs) embedded in a one-dimensional waveguide and investigate the emission statistics via the second-order correlation function $g^{(2)}(\tau)$ in a Hanbury Brown–Twiss (HBT) measurement. Each QD is modeled as a two-level system with ground state $|g\rangle$, excited state $|e\rangle$, and atomic collapse operator $\sigma = |g\rangle\langle e|$. The system dynamics are governed by the master equation in Lindblad form, containing both coherent evolution and dissipation processes.

The Hamiltonian is composed of the bare emitter's energy and their coupling via waveguide-mediated interactions. The bare Hamiltonian is given by ($\hbar = 1$)

$$H_S = -\sum_j \Delta_j \sigma_j^\dagger \sigma_j, \tag{S11}$$

where $\Delta_j = \omega_L - \omega_j$ is the detuning of the jth emitter from the laser frequency ω_L. The QDs couple through coherent, dipole-like exchange interactions mediated by the guided mode of the waveguide. The interaction Hamiltonian takes the form

$$H_I = -i\frac{\Gamma}{2}\left(e^{ik|x_1-x_2|}\sigma_1^\dagger\sigma_2 - e^{-ik|x_1-x_2|}\sigma_2^\dagger\sigma_1\right), \tag{S12}$$

where Γ is the emission rate into the waveguide, $k = 2\pi/\lambda$ is the wavevector of the guided mode, and x_1, x_2 are the positions of the QDs. The form of Eq.(S12) captures the phase-dependence $\varphi_{12} = k\ |x_1 - x_2|$ controlling the dispersive and dissipative nature of the interaction. The combinations of raising and lowering operators describe directional excitation transfer mediated by a common mode, as detailed in [7].

In a frame rotating at the excitation frequency, the system is equivalently described by the effective Hamiltonian

$$H_{eff} = \sum_{mn} (J_{mn} - i\frac{\Gamma_{mn}}{2})\sigma_n^\dagger\sigma_m. \tag{S13}$$

When $m \neq n$, Eq.(S13) describes the waveguide-mediated coupling with J_{mn} and Γ_{mn} as the coherent exchange and collective dissipation rates, determined by the relative phase, independent decay rates, and β-factors [8]

$$J_{mn} = \frac{1}{2}\sqrt{\beta_m\beta_n\gamma_m\gamma_n}\,\mathrm{Im}\{e^{i\varphi_{mn}}\} \tag{S14}$$

$$\Gamma_{mn} = \sqrt{\beta_m\beta_n\gamma_m\gamma_n}\,\mathrm{Re}\{e^{i\varphi_{mn}}\} \tag{S15}$$

When $m = n$, Eq.(S13) gives the independent contributions of each emitter with Lamb shift J and decay rate Γ.

The system is subject to dissipation via spontaneous emission, pure dephasing, and loss into unguided modes. These processes are included in the dynamics with Lindblad dissipators of the form $\mathcal{D}[L]\rho = L\rho L^\dagger - \frac{1}{2}\{L^\dagger L, \rho\}$ for system operator L. For example, a coupling efficiency β_j corresponds loss into unguided modes with a rate $(1-\beta_j)\Gamma_j$ and operator σ_j. The master equation describing the coupled dynamics of the system is then

$$\frac{d\rho}{dt} = -i[H_{eff}, \rho] + \sum_L \mathcal{D}[L]\rho, \tag{S16}$$

where the first term describes coherent evolution and the second term accounts for dissipation and additional sources of loss.

Correlation functions

The second-order correlation function $g^{(2)}(\tau)$ determines the statistical properties of a source based on fluctuations in the detected field. Using the steady-state solution of the master equation in Eq.(S16), we calculate the second-order correlation

$$g^{(2)}(\tau) = \frac{\langle E^\dagger E^\dagger(\tau) E(\tau) E\rangle}{\langle E^\dagger E\rangle^2}, \tag{S17}$$

using the quantum regression theorem [9]. We first find the steady-state solution ρ_{ss} by propagating the density matrix through a long time t according to Eq.(S16). Next, the field operator evaluated at a later time $t+\tau$ is pre- and post-multiplied by the steady-state $\hat{E}\rho_{ss}\hat{E}^\dagger$, corresponding to detection of a photon. Defining this as the initial 'pseudo-state'

density matrix, it is propagated through time τ according to the master equation. The resulting state is pre-multiplied by the earlier time operator $\hat{E}^\dagger \hat{E}$ before taking the trace to obtain

$$g^{(2)}(\tau) = \mathrm{Tr}\{\hat{E}^\dagger \hat{E}(e^{\mathcal{L}\tau}\hat{E}\rho_{ss}\hat{E}^\dagger)\}, \tag{S18}$$

where $e^{\mathcal{L}\tau}$ denotes the completely-positive trace-preserving map for the time-evolution of the system.

The form of the field operator $\hat{E}$ depends on the detection channel. For instance, the right-propagating waveguide mode is represented in terms of the system operators as

$$\hat{E}_R = \sum_j \sqrt{\beta_j \gamma_{j_R}} e^{-ikx_j} \sigma_j. \tag{S19}$$

To model realistic detector response, the computed $g^{(2)}(\tau)$ is convolved with a Gaussian response function representing the timing jitter of the detector (typically 20 ps FWHM). Additionally, background counts and imperfect signal-to-background ratios (SBR) are accounted for by modifying the correlation function as

$$g^{(2)}_{\mathrm{final}}(\tau) = 1 + \left(\frac{\mathrm{SBR}^2}{(1+\mathrm{SBR})^2}\right)\left(g^{(2)}(\tau) - 1\right). \tag{S20}$$

This approach enables detailed modeling of photon correlation experiments in systems with chiral or symmetric coupling, allowing direct comparisons with experimental data on indistinguishability, directional emission, and superradiance in photonic waveguide QED [10–12].

Parameter fitting

Using the model described above the $g^{(2)}(\tau)$ described in Fig.4 we use the following parameters to fit the curves:

Table S1: Fitting parameters for Fig.4 (main text).

**Parameter**	**Fig.4(g)**	**Fig.4(h)**	**Fig.4(i)**
Decay Time (ns^{-1})	1	2	0.25
Dephasing (ns^{-1})	25	25	25
SBR	≈ 0	≈ 0	0.1
C_1	0.8	0.8	-0.8
C_2	0.7	-0.7	0.65

The parameters used in the fitting, summarized in Table S1, include the radiative decay rate, pure dephasing rate, signal-to-background ratio (SBR), and directional coupling coefficients C_1 and C_2 that describe the amplitude of each QD's emission into the detected waveguide mode.

As shown in Figure S4, the theoretical fits reproduce the key features of the measured $g^{(2)}(\tau)$ functions across all three spin-selective combinations. Notably, the model reproduces the observed bunching across the different spin configurations. The bunching originates from interference between emission pathways in opposite directions, whereas the weak coupling of specific spin states into the counterpropagating mode limits their contribution and thereby reproducing the antibunching levels seen in single dot $g^{(2)}(\tau)$ measurements in Figure S4(a,b).

Additionally, the fitted coupling coefficients are consistent with independent measurements of the chiralities of QD1 (84%) and QD2 (74%) (shown in Figure S4(c,d)), validating the use of the directionally coupled Lindblad terms in the model. The close agreement between theory and experiment confirms that the observed spin-dependent emission contrast originates from interference effects mediated by the waveguide, as predicted by the chiral quantum optics framework.

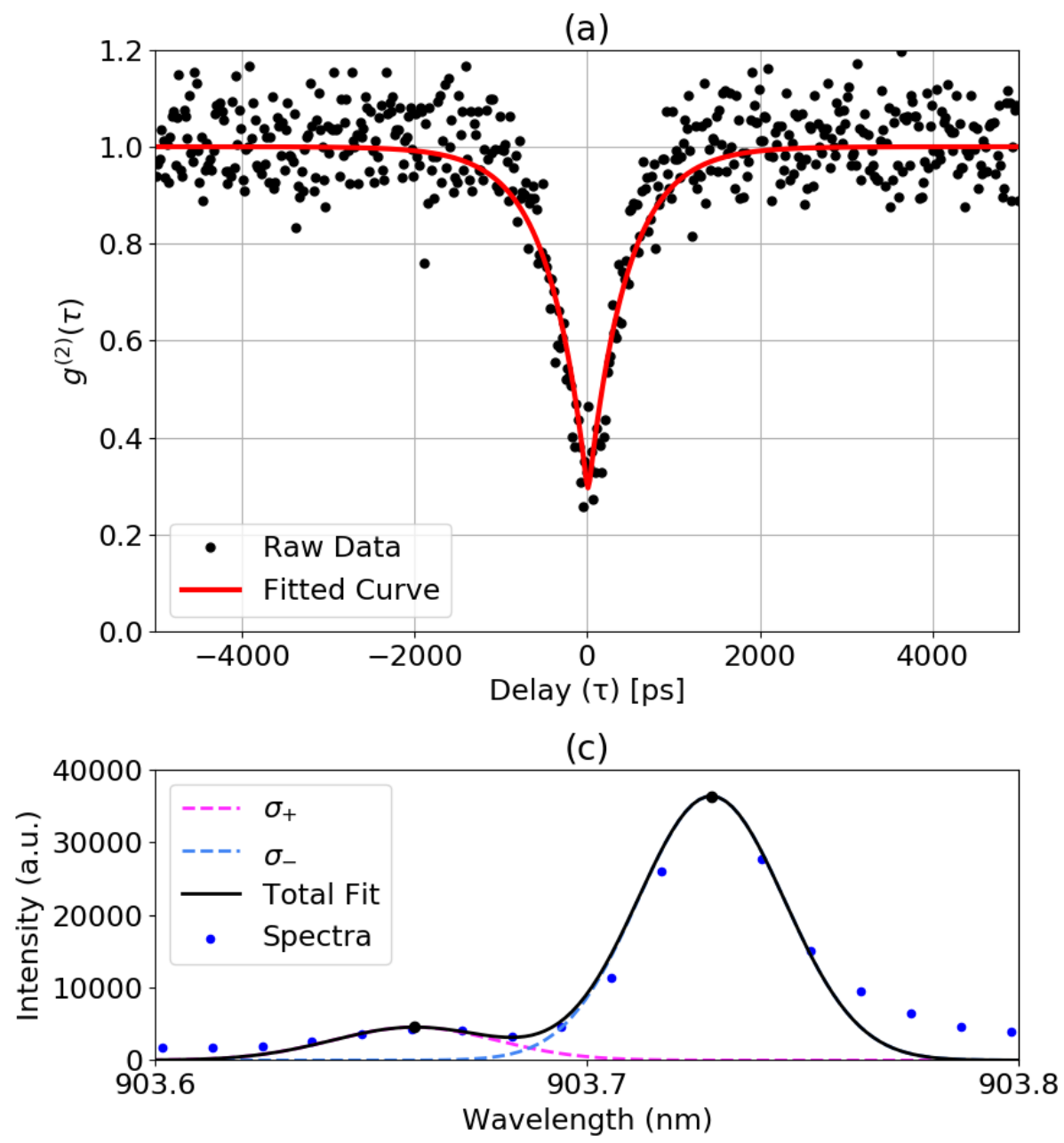

Figure S4: **Photon statistics for 2 chiral QDs.** (a,b) Second-order photon correlation functions $g^{(2)}(\tau)$ measured from QD1 (a) and QD2 (b), with fits to a single-emitter antibunching model (red line). Black points show the raw data, and red solid lines show the fitted curves.(c,d) Corresponding PL spectra of QD1 (c) and QD2 (d) under an applied magnetic field of 0.5 T. Blue points show the measured spectra, black solid lines show the total double-Gaussian fits, and the coloured component lines show the fitted Zeeman-split σ^+ and σ^- spin transitions.

SUPPLEMENTARY NOTE 6: BULK DIODE TESTS

The measurements in Figure S5 are similar to Fig.3(a–c) in the main text which involve electrically contacting the bulk diode and recording the PL under 808 nm LED excitation. Square boxes are implanted, using lines of implanted ions that very from 100 nm to 1 μm in width. By applying reverse bias, and quenching emission from the diode, we can observe the electrical isolation created by the implantation.

The QDs in this wafer emit between -0.5 V and 1.5 V. At higher biases, electroluminescence is visible, and at biases below 0.5 V emission is quenched by the large electric field. We can observe in Figure S5(b) emission is visible from all areas of the sample (except the

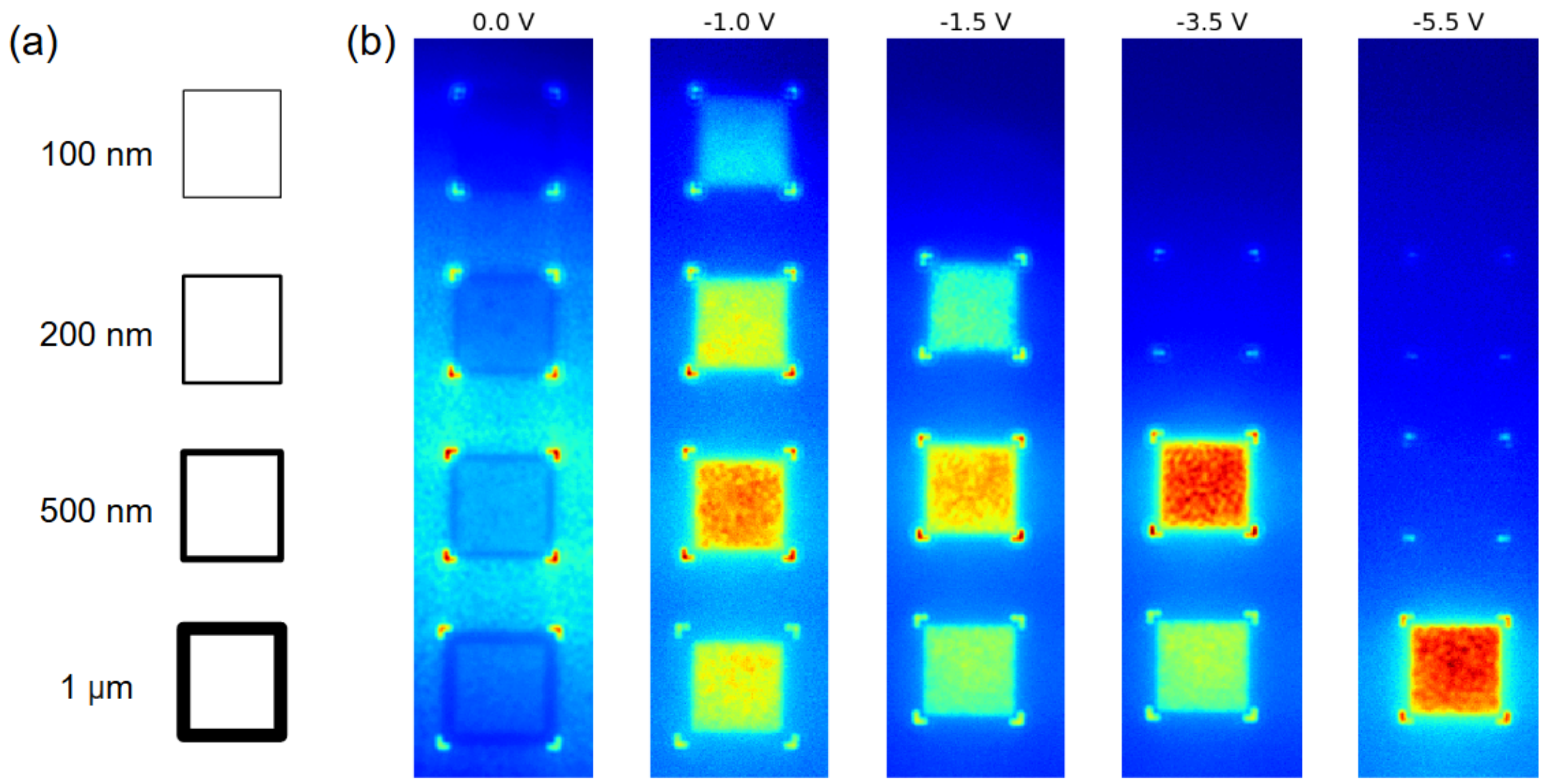

Figure S5: **Bulk diode response to implanted regions under reverse bias.** (a) Schematic representation of 5x5 μm square test regions with varying implantation widths: 100 nm, 200 nm, 500 nm, and 1 μm. (b) PL maps from a bulk diode under 808 nm LED excitation as a function of applied reverse bias (0 V to -5.5 V).

QDs directly beneath the implanted lines) when 0 V is applied to the sample, but that the emission from QDs outside the implantation areas is quenched when a voltage of -1 V is applied to the sample. This indicates that even 100 nm of implantation is enough to provide some electrical isolation.

However, when we apply larger negative biases, we can overcome the isolation caused by the implantation. At -3.5 V, the emission within the 200 nm region is quenched, and at -5.5 V the emission within the 500 nm region is quenched. In the final sample, implanted lines of 500 nm are used to provide sufficient resistance across the QD tuning range.

REFERENCES

[1] K. Chan, Y. Hui, D. Han, and G. Li, Modification of refractive index of gaas by ion implantation of oxygen, Nuclear Instruments and Methods in Physics Research Section B: Beam Interactions with Materials and Atoms **83**, 177 (1993).

[2] S. Pearton, Ion implantation doping and isolation of iii–v semiconductors, Nuclear Instruments and Methods in Physics Research Section B: Beam Interactions with Materials and Atoms **59-60**, 970 (1991).

[3] B. Bennett, R. Soref, and J. Del Alamo, Carrier-induced change in refractive index of inp, gaas and ingaasp, IEEE Journal of Quantum Electronics **26**, 113 (1990).

[4] J. R. Lowney and H. S. Bennett, Majority and minority electron and hole mobilities in heavily doped gaas, Journal of Applied Physics **69**, 7102 (1991).

[5] C. Liang, L. Vandamme, G. Leroy, and J. Gest, Resistance and 1/f noise between circular contacts on conductive thin films, European Physical Journal-applied Physics - EUR PHYS J-APPL PHYS **53** (2011).

[6] S. M. Sze and K. K. Ng, *Physics of Semiconductor Devices*, 3rd ed. (Wiley-Interscience, 2006) p. 189.

[7] A. Gonzalez-Tudela, D. Martin-Cano, E. Moreno, L. Martin-Moreno, C. Tejedor, and F. J. Garcia-Vidal, Entanglement of two qubits mediated by one-dimensional plasmonic waveguides, Phys. Rev. Lett. **106**, 020501 (2011).

[8] A. Tiranov, V. Angelopoulou, C. J. van Diepen, B. Schrinski, O. A. D. Sandberg, Y. Wang, L. Midolo, S. Scholz, A. D. Wieck, A. Ludwig, A. S. Sørensen, and P. Lodahl, Collective super- and subradiant dynamics between distant optical quantum emitters, Science **379**, 389 (2023), https://www.science.org/doi/pdf/10.1126/science.ade9324.

[9] H. J. Carmichael, *An Open Systems Approach to Quantum Optics*, Lecture Notes in Physics, Vol. m18 (Springer, 1993).

[10] M. J. Collett and C. W. Gardiner, Squeezing of intracavity and traveling-wave light fields produced in parametric amplification, Physical Review A **30**, 1386 (1984).

[11] M. Arcari, I. Söllner, A. Javadi, S. Lindskov Hansen, S. Mahmoodian, J. Liu, H. Thyrrestrup,

E. H. Lee, J. D. Song, S. Stobbe, and P. Lodahl, Near-unity coupling efficiency of a quantum emitter to a photonic crystal waveguide, Phys. Rev. Lett. **113**, 093603 (2014).

[12] I. Söllner, S. Mahmoodian, S. L. Hansen, L. Midolo, A. Javadi, G. Kiršanskė, T. Pregnolato, H. El-Ella, E. H. Lee, J. D. Song, S. Stobbe, and P. Lodahl, Deterministic photon-emitter coupling in chiral photonic circuits, Nature Nanotechnology **10**, 775 (2015).
